# The Prognostic Value of Anti-PLA2R Antibodies Levels in Primary Membranous Nephropathy

**DOI:** 10.3390/ijms24109051

**Published:** 2023-05-21

**Authors:** Olga Lesya Kukuy, Ron Cohen, Boris Gilburd, Eleanor Zeruya, Talia Weinstein, Timna Agur, Dganit Dinour, Pazit Beckerman, Alexander Volkov, Johnatan Nissan, Tima Davidson, Howard Amital, Yehuda Shoenfeld, Ora Shovman

**Affiliations:** 1 Institute of Nephrology and Hypertension, Sheba Medical Center, Ramat Gan 5265601, Israel; 2 Sackler Faculty of Medicine, Tel-Aviv University, Tel-Aviv 6997801, Israel; 3 Zabludowicz Center for Autoimmune Diseases, Sheba Medical Center, Ramat Gan 5265601, Israel; 4 Department of Nephrology, Tel Aviv-Sourasky Medical Center, Tel Aviv 6423906, Israel; 5 Department of Nephrology and Hypertension, Rabin Medical Center, Petah-Tikva 4941492, Israel; 6 Institute of Pathology, Sheba Medical Center, Ramat Gan 5265601, Israel; 7 Department of Nuclear Medicine, Sheba Medical Center, Ramat Gan 5265601, Israel; 8 Department of Medicine ‘B’, Sheba Medical Center, Ramat Gan 5265601, Israel; 9 Laboratory of the Mosaics of Autoimmunity, Saint Petersburg State University, 199034 Saint-Petersburg, Russia

**Keywords:** anti-PLA2R antibodies, primary membranous nephropathy, ELISA

## Abstract

Anti-PLA2R antibodies (Ab) are a diagnostic and prognostic biomarker in primary membranous nephropathy (PMN). We assessed the relationship between the levels of anti-PLA2R Ab at diagnosis and different variables related to disease activity and prognosis in a western population of PMN patients. Forty-one patients with positive anti-PLA2R Ab from three nephrology departments in Israel were enrolled. Clinical and laboratory data were collected at diagnosis and after one year of follow-up, including serum anti-PLA2R Ab levels (ELISA) and glomerular PLA2R deposits on biopsy. Univariable statistical analysis and permutation-based ANOVA and ANCOVA tests were performed. The median [(interquartile range (IQR)) age of the patients was 63 [50–71], with 28 (68%) males. At the time of diagnosis, 38 (93%) of the patients had nephrotic range proteinuria, and 19 (46%) had heavy proteinuria (≥8 gr/24 h). The median [IQR] level of anti-PLA2R at diagnosis was 78 [35–183] RU/mL. Anti-PLA2R levels at diagnosis were correlated with 24 h proteinuria, hypoalbuminemia and remission after one year (*p* = 0.017, *p* = 0.003 and *p* = 0.034, respectively). The correlations for 24 h proteinuria and hypoalbuminemia remained significant after adjustment for immunosuppressive treatment (*p* = 0.003 and *p* = 0.034, respectively). Higher levels of anti-PLA2R Ab at diagnosis in patients with active PMN from a western population are associated with higher proteinuria, lower serum albumin and remission one year after the diagnosis. This finding supports the prognostic value of anti-PLA2R Ab levels and their possible use in stratifying PMN patients.

## 1. Introduction

Membranous nephropathy (MN) is one of the main causes of nephrotic syndrome in adults. In 75–80% of patients, MN occurs in the absence of identifiable causes and is therefore classified as primary MN (PMN) [1,2]. In contrast, secondary MN (SMN) accounts for approximately 20–25% of MN cases and occurs due to a variety of other illnesses including malignancies, autoimmune systemic diseases, drug reactions and infections [3].

M-type phospholipase A2 receptor (PLA2R) is an antigen [4] that plays an important role in the pathogenesis and disease progression of PMN [1,5,6]. Therefore, the presence of serum anti-PLA2R antibodies (Ab) and/or glomerular PLA2R deposits may be useful in differentiating between PMN and SMN [5]. Different studies reported that anti-PLA2R Ab are highly specific to PMN, although there are rare reports of anti-PLA2R-positive MN associated with sarcoidosis, hepatitis B and C, human immunodeficiency virus, and cancer [3].

The clinical relevance of anti-PLA2R Ab in PMN has been addressed in several studies [7,8,9,10,11,12,13,14]. A number of studies reported that presence of serum anti-PLA2R Ab and high levels of anti-PLA2R Ab in PMN patients correlate with different markers of disease activity, such as serum creatinine, serum albumin, proteinuria and estimated glomerular filtration rate (eGFR) [7,8,9,10]. In contrast, other studies failed to show a significant correlation between clinical and the immunological disease activities, particularly at the beginning of an overt disease [11,12,13,14,15].

The clinical outcome of PMN patients is variable, ranging from spontaneous remission to end-stage renal disease (ESRD) [16]. Thus, identification of reliable prognostic biomarkers is crucial for risk stratification and selection of appropriate therapeutic strategy. In this regard, the prognostic value of anti-PLA2R Ab in PMN patients remains a topic of ongoing research.

Recently, several studies and three meta-analyses have assessed the relationship between anti-PLA2R expression and clinical outcome in PMN patients [8,17,18,19,20,21,22,23,24]. Collective results from both meta-analyses showed that, in general, elevated anti-PLA2R levels are associated with a worse prognosis in patients with PMN [22,23,24]. However, several studies included in the meta-analyses were heterogeneous, with differing ethnicity, number of patients, and anti-PLA2R testing methods, and incomplete data regarding treatment. Additionally, the prevalence of anti-PLA2R Ab in PMN patients is variable, ranging between 30% and 89%, and depending mainly on ethnicity and the detection method used [24]. The majority of studies were performed in Asia, and a relatively low number of studies were performed in Western countries. Therefore, more data are required regarding the prognostic utility of anti-PLA2R Ab in Western populations. In addition, the presence of glomerular PLA2R deposits was evaluated as a possible prognostic indicator, and in one study the deposits were associated with disease relapse [25].

In the present study, we assessed the relationship between the levels of anti-PLA2R Ab at diagnosis and different clinical and laboratory variables related to disease activity and prognosis.

## 2. Results

One hundred and sixty-five patients tested positive for serum anti-PLA2R Ab during the study period. Clinical data at baseline were available for forty-four patients that were treated at the three medical centers participating in this study. Two patients were excluded due to biopsy findings of amyloidosis and minimal change in disease, and one patient was excluded due to a diagnosis of Hodgkin lymphoma.

Forty-one patients were included in the final analysis. In 34 patients, the time of diagnosis was determined based on serum anti-PLA2R positivity, and in 7 patients the time of diagnosis was determined by biopsy results that showed the presence of MN with glomerular PLA2R deposits. For these 7 patients, the level of anti-PLA2R antibodies at diagnosis was not available and assessed at a later timepoint. Relevant data one year after the diagnosis were available for 32 patients, except for leg edema and hypertension.

The median [interquartile range (IQR)] age was 63 [50–71], and 28 (68%) of them were male. At the time of diagnosis, 39 (95%) of the patients had nephrotic range proteinuria, and 19 (46%) had heavy proteinuria (≥8 gr/24 h). Leg edema and hypertension at the time of diagnosis were reported in 33 (80%) patients and 26 (63%) patients, respectively. Two patients (5%) had chronic kidney disease (CKD) stage ≥4 at the time of diagnosis.

All patients were treated with angiotensin-converting-enzyme inhibitors (ACE-I) or angiotensin II receptor blockers (ARB).

Within one year after diagnosis, 24 (59%) of the patients received IST. This treatment included Ponticelli protocol (8 patients, 20%), rituximab (6, 15%), glucocorticoids (3, 7%) and tacrolimus (1, 2%). Additionally, six patients (15%) received more than one line of IST. One year after the diagnosis, 12 patients (38%) had achieved remission (partial and complete), and 3 additional patients had progressed to CKD stage ≥4.

Laboratory data at baseline and one year after the diagnosis are summarized in Table 1. The median [IQR] levels of anti-PLA2R at diagnosis was 78 [35–183] RU/mL. One year afterwards, anti-PLA2R levels were available for 13 patients, with median [IQR] levels being 33 [3–106] RU/mL.

Thirty-two patients underwent renal biopsy, which showed typical characteristics of MN at the time of diagnosis. Staining for glomerular PLA2R deposits was performed in 22 patients, and staining was positive in 20 (91%) of them.

The relationship between serum anti-PLA2R levels at diagnosis and the various clinical and laboratory variables are shown in Table 2. No correlation was found between anti-PLA2R levels and age or gender.

**Figure 1:**
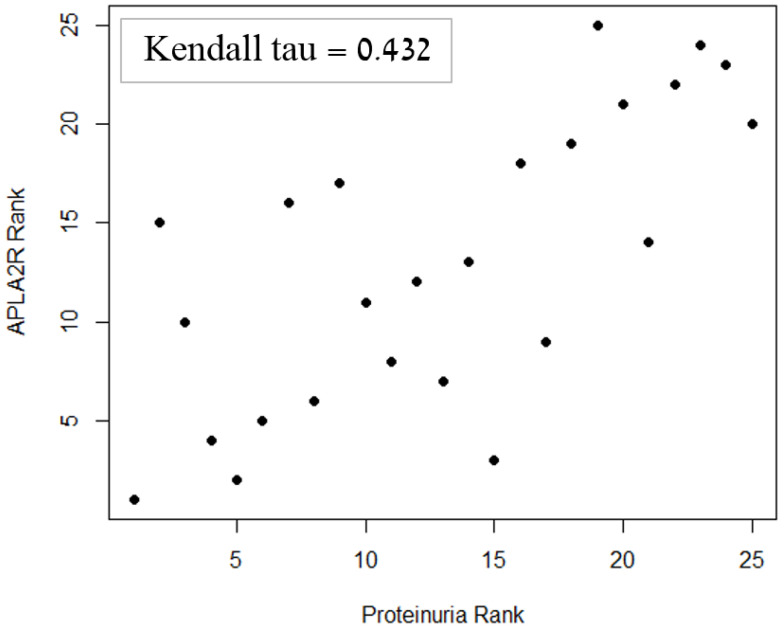
Relationship between serum anti-PLA2R antibodies level at diagnosis and 24 h proteinuria one year after the time of diagnosis. Patients were ranked by the level of anti-PLA2R antibodies at diagnosis and 24 h proteinuria after one year, and a direct correlation was found between the ranks.

**Figure 2:**
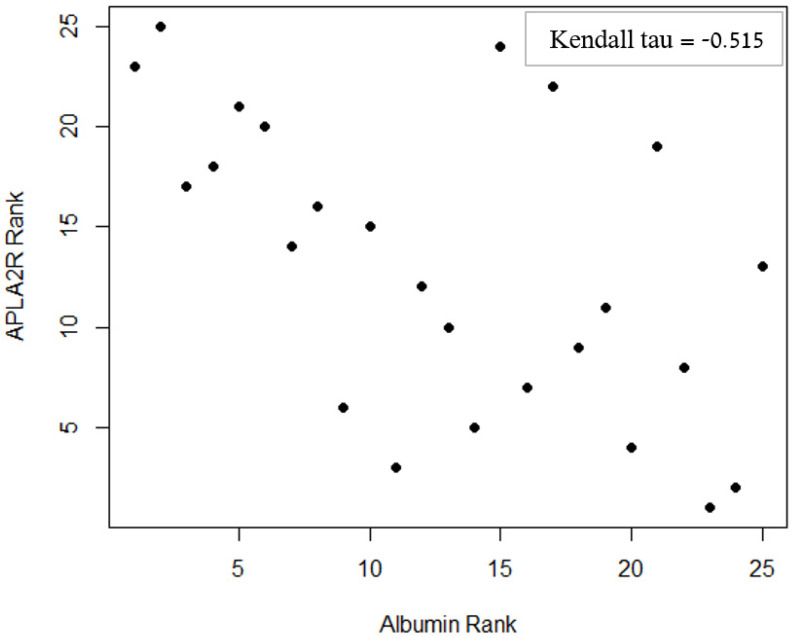
Relationship between serum anti-PLA2R antibodies level at diagnosis and serum albumin one year after the time of diagnosis. Patients were ranked by the level of anti-PLA2R antibodies at diagnosis and serum albumin after one year, and an inverse correlation was found between the ranks.

A significant correlation was found between anti-PLA2R levels at the time of diagnosis and serum albumin after one year (*p* = 0.003, Figure 1), as well as proteinuria levels after one year (*p* = 0.017, Figure 2).

After one year of follow-up, albumin and proteinuria were moderately correlated with each other (τ = −0.57). Due to this collinearity, adjustment for IST was performed separately for albumin and proteinuria, using two permutation-based ANCOVA tests with IST as an additional factor. Even after this adjustment, albumin and proteinuria remained significantly correlated with anti-PLA2R levels at the time of diagnosis (*p* = 0.003 and *p* = 0.034, respectively).

In addition, lower anti-PLA2R levels were correlated with higher rates of remission after one year (*p* = 0.034). After adjustment for IST by permutation-based ANOVA test, this correlation was not significant (*p* = 0.096).

## 3. Discussion

PMN has an unpredictable course, varying from spontaneous remission to persistent proteinuria and ESRD in the absence of appropriate IST. Thus, the recognition of prognostic biomarkers is especially important for risk-stratification in these patients [16]. Previously, elevated serum creatinine level at the time of diagnosis, persistent high-grade proteinuria and rapid rate of deterioration of renal function over 6 months have been identified as clinical predictors for poor renal outcome in PMN patients [26]. However, although severe proteinuria and high serum creatinine levels at different follow-up times may reflect the disease severity, they cannot differentiate between active disease and non-active disease with irreversible structural kidney damage [27].

In recent years, the integration of clinical biomarkers and serological biomarkers, such as anti-PLA2R Ab and other novel autoantibodies, is increasingly utilized for the determination of disease prognosis and to guide the appropriate treatment strategy [19,21,27,28]. In the current study, higher levels of anti-PLA2R Ab at the time of diagnosis in patients with active PMN were directly associated with proteinuria and inversely correlated with serum albumin level measured one year after the time of diagnosis. This correlation remained significant after adjustment for IST, similarly to a recent report that evaluated PMN patients that received different ISTs [29], which reinforces the prognostic value of these antibodies. Our results are also consistent with other reports that showed that anti-PLA2R Ab levels at baseline were significantly correlated with the severity of proteinuria that appeared later in the course of the disease and were associated with disease severity [30,31]. Moreover, additional studies found that high baseline levels of anti-PLA2R Ab are associated with the development of nephrotic syndrome in patients with non-nephrotic range proteinuria [27] and with a progressive deterioration in renal function [32,33,34].

The present study cohort included patients with severe active PMN. Most of them had nephrotic range proteinuria and approximately half of them had heavy proteinuria. A total of 59% of the patients required IST within one year after the diagnosis and 38% achieved clinical remission after one year. In our cohort, a significant association was found between lower serum anti-PLA2R levels and clinical remission, but this association was not significant when IST was added as an independent factor. In this regard, a recent meta-analysis showed that patients with higher anti-PLA2R Ab levels at the initiation of treatment had a lower probability of clinical remission, especially in the Asian population subgroup [24].

CKD stage ≥4 was observed in 2 patients at diagnosis and in an additional 3 patients at the one-year time point, and all of them were in the highest tertile with regard to anti-PLA2R levels at diagnosis. Previously, the presence of anti-PLA2R Ab in patients with PMN was found to be an independent risk factor for developing CKD stage ≥3 [30]. Furthermore, one report showed that a higher titer of anti-PLA2R Ab is correlated with higher risk for CKD stage ≥4 [18].

In the present study, no correlation was observed between the levels of anti-PLA2R Ab and clinical parameters of disease activity at the time of diagnosis. Prior data regarding this possible association are controversial. Several studies found that patients with higher levels of anti-PLA2R Ab at diagnosis had a higher level of clinical disease activity [7,8,9,10], while others failed to find any statistically significant association [11,12,13,14]. This disparity may be explained by the gap between the appearance of anti-PLA2R Ab and the emergence of a clinically manifest PMN, which may be as long as 8 months [11,12]. In addition, it has been observed that an increase or decrease in anti-PLA2R levels precedes an increase or decrease in proteinuria, respectively [7]. Although the levels of anti-PLA2R were tested at the beginning of the disease in the current study, all of our patients were treated with ACE-I or ARBs at this time point, which may have affected the severity of proteinuria and influenced the possible correlation.

Anti-PLA2R Ab represents a mechanistic biomarker, and several reports have established the role of anti-PLA2R Ab in the pathogenesis of PMN [1,6]. The correlation between anti-PLA2R Ab levels and severity of proteinuria after one year, but not at baseline, may be explained by the accumulating damage to the filtration barrier induced by the binding of circulating anti-PLA2R Ab to the PLA2R antigen on podocytes.

In our cohort, three patients with positive anti-PLA2R Ab had causes for secondary MN and were thus excluded from this study. Anti-PLA2R autoimmunity is considered rare in secondary MN, but such a coincidental association had been previously described in patients with sarcoidosis and hepatitis B [3,25].

Our study has several limitations. Firstly, our cohort size is relatively small. It should be noted that tests for anti-PLA2R Ab are relatively new, and they are performed in only two medical centers in Israel. Therefore, many patients who perform anti-PLA2R testing in the Sheba Medical Center were treated in other medical centers; therefore, clinical data are not available for them. Another limitation is the retrospective design of the study. In this design, it is difficult to create a uniform database for all patients that were recruited from three different medical centers.

## 4. Materials and Methods 

### 4.1. Study Design and Population

This is a multi-center retrospective study that included patients from the nephrology departments of three medical centers: Sheba Medical Center, Tel Aviv Sourasky Medical Center and Rabin Medical Center.

The study was conducted on patients that were found to have serum anti-PLA2R Ab in the diagnostic laboratory of the Zabludowicz Center for Autoimmune Diseases in Sheba Medical Center between 2014 and June 2019. Patients with a proven diagnosis of MN and available demographic, clinical, laboratory and histopathologic data were selected for further analysis. Patients with other renal diseases and patients with possible causes of secondary MN were excluded.

### 4.2. Collection of Data

We collected demographic, clinical, laboratory and histopathologic data from the computerized medical records of three medical centers.

The demographic variables included age and gender. Clinical variables included presence of leg edema and hypertension (≥130 systolic or ≥80 diastolic). Laboratory and histopathologic variables that were assessed consisted of serum creatinine, serum albumin, 24 h urine proteinuria, estimated glomerular filtration (eGFR), levels of serum anti-PLA2R Ab and the presence of glomerular PLA2R deposits on kidney biopsy.

Data were extracted retrospectively from medical records at the time of diagnosis and one year after the diagnosis. The time of diagnosis was determined by the presence of positive anti-PLA2R Ab in patients with clinical suspicion of MN or by biopsy findings suggesting MN with PLA2R deposits that were subsequently confirmed by the presence of anti-PLA2R Ab. Data that were recorded within 3 months of the relevant time point were included in the analysis.

Clinical remission was defined as proteinuria of <3.5 g/day and was further divided into complete remission (proteinuria < 0.3 g/day) and partial remission (proteinuria between 0.3 g/day and 3.5 g/day). eGFR was calculated according to the Chronic Kidney Disease Epidemiology Collaboration (CKD-EPI) formula [35].

### 4.3. Statistical Analysis

The variables were classified into continuous and categorical. The normality of continuous variables was visually assessed by a histogram and by the Shapiro–Wilk test. Continuous variables were presented as median (IQR). Categorical variables were presented as percentages.

We evaluated the relationship between serum anti-PLA2R Ab levels and demographic, clinical and laboratory variables at diagnosis and one year afterwards.

Due to the non-normal distribution of serum anti-PLA2R Ab levels, non-parametric tests were preferred. Thus, the correlation between continuous variables was assessed using Kendall’s tau. The relationship between continuous variables and categorical variables was assessed by Mann–Whitney U test. In order to account for the effects of immunosuppressive treatment (IST), statistically significant variables from the previous step were entered with IST into a multivariable permutation-based analysis of variance (ANOVA) or covariance (ANCOVA). Adjustment for multiple hypotheses testing was performed using the Benjamini–Hochberg method and variables with adjusted *p* values < 0.05 were considered significant. Statistical analyses were performed with SPSS (version 25) and the lmPerm package (version 2.1.0) in R (version 4.0.2).

### 4.4. Detection of Serum Anti-PLA2R Antibodies

Serum anti-PLA2R antibody was measured by an ELISA test employing previously validated commercial kits (EUROIMMUN, Lubeck, Germany) according to the manufacturer’s instructions [36]. A result of <14 relative units (RU/mL) was considered as negative, and a result of ≥15 RU/mL was considered positive, according to the manufacturer’s specifications.

### 4.5. PLA2R Immunohistochemistry from Renal Biopsy

Renal biopsies from patients with PMN were stained for the PLA2R antigen. Formalin-fixed, paraffin-embedded (FFPE) blocks were sectioned at 3.5 µm and a positive control was added on the right edge of the slides. The slides were warmed up to 60 °C for 1 h and were processed by a fully automated protocol on a Benchmark Ultra staining module according to the manufacturer’s recommendations (Ventana medical Systems, Tucson, AZ, USA). Briefly, after sections were dewaxed and rehydrated, a Mild CC1 Benchmark Ultra pretreatment for antigen retrieval (Ventana Medical Systems Inc., USA) was selected for PLA2R (Diluted 1:1000, Sigma-Aldrich, HPA012657, St Louis, USA). Anti-PLA2R antibodies were detected with UltraView DAB Detection Kit (Ventana medical Systems, Tucson, AZ, USA). Sections were incubated for 40 min with anti-PLA2R antibodies and counterstained with Hematoxylin II (Ventana medical Systems, Tucson, AZ, USA). After the run on the automated stainer was completed, the slides were dehydrated in graded ethanols (70%, 96%, and 100%). Before cover-slipping, sections were cleared in Xylene and mounted with Entellan.

## 5. Conclusions

We demonstrated that higher levels of anti-PLA2R Ab at diagnosis in patients with active PMN are directly associated with proteinuria and inversely correlated with serum albumin measured one year following the time of diagnosis. Lower levels of anti-PLA2R at diagnosis were correlated with higher rates of remission, but this correlation was not independent of the effect of IST. This finding supports the prognostic value of anti-PLA2R Ab levels, which should be taken into account in the management of PMN patients.

## Figures and Tables

**Table 1:** Laboratory characteristics of the study population.

Variable	Median	IQR
**Baseline (*n* = 41)**		
Creatinine (mg/dL)	0.89	0.73–1.13
Albumin (g/dL)	2.70	2.25–3.00
Proteinuria (g/24-h)	7.80	6.20–10.90
Anti-PLA2R (RU/mL)	78	35–183
eGFR (mL/min)	89	64–103
**One year after diagnosis (*n* = 32)**		
Creatinine (mg/dL)	0.98	0.82–1.25
Albumin (g/dL)	3.05	2.48–3.40
Proteinuria (g/24-h)	4.85	2.01–11.55
eGFR (mL/min)	78	55–103

**Table 2:** Relationship between serum anti-PLA2R levels at diagnosis and laboratory variables.

Kendall Correlation				
Variable	Kendall’s Tau		*p*-Value	Adjusted *p*-Value
**At diagnosis**				
Creatinine	0.102		0.406	0.575
Albumin	−0.142		0.252	0.444
24 h Proteinuria	−0.054		0.667	0.810
eGFR	−0.005		0.976	0.976
**One year after diagnosis**				
Creatinine	−0.165		0.261	0.444
Albumin	−0.515 *		<0.001 *	0.003 *
24 h Proteinuria	0.432 *		0.003 *	0.017 *
eGFR	0.167		0.252	0.444
**Mann–Whitney U-test**				
Variable	Median [IQR] Anti-PLA2R levels		*p*-value	Adjusted *p*-value
Hypertension (at diagnosis)	Yes	88 [42–257]	0.337	0.521
	No	66 [29–120]		
Leg edema (at diagnosis)	Yes	81 [32–190]	0.873	0.969
	No	76 [49–201]		
Remission (after 1 year)	Yes	27 [23–87]	0.009	0.034 *
	No	91 [58–278]		

* *p* value < 0.05.

## Data Availability

The data presented in this study are available on request from the corresponding author.
